# The effect of exercise motivation on college students’ self-efficacy: the mediating roles of leisure satisfaction and mental toughness

**DOI:** 10.3389/fpsyg.2024.1465138

**Published:** 2024-10-09

**Authors:** Chengfeng Yu, Zhaohong Zeng, Aochuan Xue, Qianjin Wu

**Affiliations:** ^1^School of Physical Education, Shandong University, Jinan, Shandong, China; ^2^School of Sports and Health, Zunyi Medical University, Zunyi, Guizhou, China; ^3^School of Physical Education and Health, East China Normal University, Shanghai, China

**Keywords:** exercise motivation, self-efficacy, leisure satisfaction, mental toughness, structural equation modeling

## Abstract

**Background:**

The theory of motivation suggests that individual motivation is moderately stimulated to drive individuals to engage in the behaviors for which they are motivated. It is therefore that the moderate stimulation of exercise motivation will likely lead to the enhancement of college students’ participation in exercise.

**Objective:**

Investigate the effect of exercise motivation on college students’ self-efficacy, reveal the mediating role of leisure satisfaction and mental toughness, and provide empirical evidence improving college students’ self-efficacy through exercise motivation.

**Methods:**

A stratified whole-sample approach was employed to survey 715 college students, based on a correlation table with good reliability and validity. Scale items were designed to collect subjects’ exercise motivation, self-efficacy, leisure satisfaction, and mental toughness in the study context. Mediation effect analyses were carried out using SPSS and AMOS.

**Results and conclusion:**

A significant positive effect of exercise motivation on self-efficacy (*β* = 0.18, *p* < 0.001), leisure satisfaction (*β* = 0.50, *p* < 0.001), and mental toughness (*β* = 0.45, *p* < 0.001). Leisure satisfaction and mental toughness had a significant positive effect on self-efficacy (*β* = 0.40, *p* < 0.001; *β* = 0.30, *p* < 0.001). Furthermore, leisure satisfaction and mental toughness significantly mediated in exercise motivation and self-efficacy. In conclusion, our findings further explored the effects of exercise motivation on college students’ self-efficacy and revealed the mediating roles of leisure satisfaction and mental toughness.

## 1 Introduction

Motivation is the intrinsic psychological tendency or internal drive that inspires and sustains an individual’s actions and directs them toward a certain goal, and it is the intrinsic motivation that determines behavior (Bandhu et al., 2024; Cook and Artino, 2016). Intrinsic motivation, in particular, plays a significant role in determining human behavior. Exercise motivation (EM) can be defined as the intrinsic or extrinsic motivation that prompts individuals to engage in sports activities (Barkoukis et al., 2024; Zhu et al., 2024). This motivation can be derived from the individual’s intrinsic needs, desires, or values, or it can be influenced by external factors such as rewards, social pressures, or expectations (Deci and Ryan, 2013; Deci et al., 2001). Motivation derived from the pursuit of achievement, health, socialization, and other factors can significantly impact the motivation, persistence, and quality of performance of individuals engaged in sporting activities.

Self-efficacy can be defined as an individual’s subjective judgment about their capacity to perform a specific action to attain a desired outcome (Schunk, 1995; Bandura, 1991). It encapsulates the individual’s belief in their capability to execute a given action in order to achieve a particular purpose. The self-efficacy theory posits that self-efficacy can influence behavior, and that behavior can also influence self-efficacy (Jia and Wang, 2024; McLaren et al., 2024; McLaren et al., 2023). Furthermore, individuals with high self-efficacy demonstrate elevated levels of self-confidence and greater adherence to behavioral expectations in the context of diverse challenges and difficulties (Rafiei et al., 2024). The efficacy beliefs of college students are shaped by a combination of past experiences, personal attributes, and social support, which in turn influence their engagement in sporting activities. In examining the impact of sports motivation on self-efficacy, it is essential to consider the potential mediating influence of an individual’s intricate intrinsic motivational factors.

A study conducted by Kavussanu and Roberts (1996) on college students who were beginning to play tennis revealed that, among males, mastery of the motivational climate was a significant predictor of self-efficacy. In contrast, among females, the strongest predictor was motivation for performance. In a survey conducted by Pauline (2013) of 871 undergraduate students, it was found that gender differences in self-efficacy were equally influential with regard to college students’ physical activity behaviors and motivation to exercise. Males were observed to be more confident and self-protective in undertaking challenging physical activity, whereas females needed help from others in developing an exercise regimen in order to be able to further exercise (Pauline, 2013). In light of the aforementioned theories and related literature, this study puts forth hypothesis:

*H1*: Exercise motivation has a significant positive effect on college students’ self-efficacy.

Leisure satisfaction through exercise activities can be defined as a positive emotional state resulting from the engagement in or selection of leisure activities (Beard and Ragheb, 1980; Teno et al., 2024). It represents the extent to which individuals experience satisfaction with their leisure experience or situation. It is evident that physical and recreational sports play an instrumental role in shaping the self-perception and psychological well-being of college students. These activities not only bolster physical vitality but also facilitate social interactions and mental growth. A study conducted by Lee, Gun-Chur et al. revealed a positive correlation between college students’ motivation to engage in sports and their overall satisfaction with their college experience. This was determined through a comprehensive survey and analysis of 419 college students who participated in recreational sports (Lee, 2019). The study conducted by Wu et al. (2021) on a sample of secondary school students in Chengdu, Sichuan Province, China, demonstrated a significant positive correlation between leisure motivation and leisure satisfaction. In light of the aforementioned evidence, this paper proposes the research hypothesis:

*H2*: Exercise motivation significantly and positively effects college students’ leisure satisfaction.

*H3*: Leisure satisfaction significantly and positively effects college students’ self-efficacy.

*H4*: Leisure satisfaction mediates the influence of exercise motivation on college students’ self-efficacy.

Mental toughness is a dynamic process in which life events, such as stress and adversity, act simultaneously with protective factors (Richardson et al., 1990; Li et al., 2024). In the context of competitive sports, mental toughness is regarded as a pivotal factor in an athlete’s capacity to navigate the inherent pressures of competition and rebound from setbacks. Athletes who demonstrate elevated levels of mental toughness tend to exhibit superior performance under pressure and demonstrate accelerated recovery from suboptimal outcomes. Prior empirical studies have demonstrated a robust positive correlation between mental toughness and self-efficacy (Ali et al., 2024). Athletes with higher self-efficacy are more likely to develop resilience, which enables them to cope more effectively with stress and challenges. Furthermore, participation in enjoyable leisure activities (higher leisure satisfaction) has been demonstrated to enhance an individual’s capacity to effectively cope with stressors encountered during sporting activities (Freire and Teixeira, 2018; Pressman et al., 2009). This may be attributed to the fact that individuals who report high levels of leisure satisfaction tend to exhibit superior mental toughness (Terzi et al., 2024). In light of the above, this paper puts forth the research hypothesis:

*H5*: Exercise motivation significantly and positively effects mental toughness in college students.

*H6*: Mental toughness significantly and positively effects self-efficacy among college students.

*H7*: Mental toughness mediates the effect of motivation on self-efficacy among college students.

**Figure 1:**
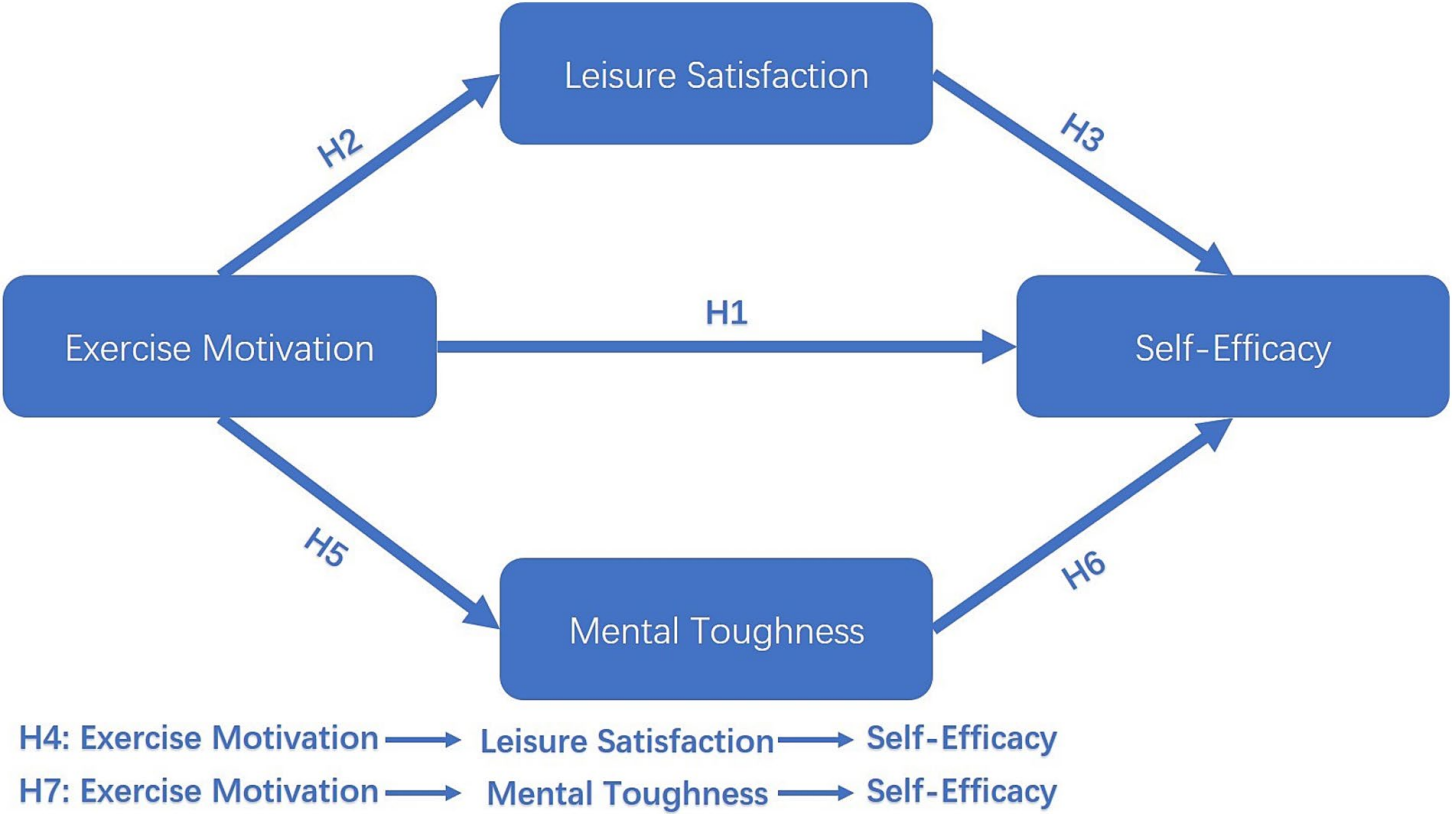
Conceptual model proposed in this study.

In recent years, researchers at home and abroad have engaged in impassioned debate regarding the motivations of college students to participate in sports. However, it is equally important to focus on the reasons for these motives and their impact on college students, both physically and psychologically. Accordingly, this study employs college students as the research object to investigate the impact of sports motivation on college students’ self-efficacy and to elucidate the mediating role of leisure satisfaction and human mental toughness. The mediation model, which synthesizes the research hypotheses, is presented in Figure 1.

## 2 Methods

### 2.1 Participants

The participants in this study were selected from among students enrolled in four undergraduate colleges in Shandong Province. A total of 720 questionnaires were distributed online, and 715 valid questionnaires were obtained after data cleaning and the removal of invalid questionnaires. The questions of the Exercise Motivation Scale, Leisure Satisfaction Scale, Mental Toughness Scale, and Self-Efficacy Scale were based on the Likert five-point scale, with 1 indicating a strong disagreement and 5 indicating strong agreement. The quantitative data obtained were employed as the foundation for the empirical analyses.

### 2.2 Data collection

#### 2.2.1 Exercise Motivation Scale

In order to meet the requirements of this study, the Behavioral Motivation Scale for Physical Education (BRPEQ) was modified and subsequently employed to investigate motivation for physical education. The BRPEQ is based on the Dutch SREQ-1, which was revised and renamed BRPEQ by various scholars in subsequent studies. As illustrated in Table 1, the scale was employed to examine the motivation of students to engage in physical education across five dimensions, encompassing internal motivation, identity regulation, external regulation, and a lack of motivation (Biese et al., 2024). The scale was scored on a 5-point Likert scale, with 1 indicating a high level of non-compliance and 5 indicating a high level of compliance.

**Table 1:** Exercise Motivation Scale.

Serial number	Measurement items
EM1	Because it allows me to learn more about my favorite sport.
EM2	Because of the pleasure I get from learning sports skills, I’ve never tried before.
EM3	Because I feel a great sense of personal satisfaction when I master certain difficult sports techniques.
EM4	Because of the pleasure I feel in identifying and correcting my own shortcomings.
EM5	Because of the joy I felt in the exciting experience of the sport.
EM6	Because I love the feeling of being totally immersed in a sport.

#### 2.2.2 Leisure Satisfaction Scale

The Leisure Satisfaction Scale (LSS) is a psychometric instrument designed to assess the degree of satisfaction derived by an individual from engaging in leisure activities. The scale was initially developed by scholars such as Beard (Beard) and Ragheb (Ragheb) and has undergone multiple revisions and validations (Trottier et al., 2002). In order to align with the specific requirements of this study, the Leisure Satisfaction Scale was modified and subsequently employed in the survey, with reference to the adapted and validated version of the scale in the Chinese context. The content validity of the questionnaire was evaluated through an expert survey, and the results demonstrated a high level of validity. As shown in Table 2, the scale comprises 11 items, which have been grouped into six principal dimensions: psychological, educational, social, relaxation, physical, and aesthetic. The scale was scored on a 5-point Likert scale, with 1 indicating a response that was “very non-compliant” and 5 indicating a response that was “very compliant.”

**Table 2:** Leisure Satisfaction Scale.

Serial number	Measurement items
LS1	My sports and leisure activities give me confidence.
LS2	My sports and leisure activities give me a sense of achievement.
LS3	My sports and leisure activities increase my awareness of my surroundings.
LS3	My sports and leisure activities provide me with the opportunity to try new things.
LS5	My sports and leisure activities help me to understand others.
LS6	I interact socially with others through sports and leisure activities.
LS7	My sports and leisure activities help me to find my soul mate.
LS8	My sports and leisure activities help me to relax.
LS9	My sports and leisure activities make me happy.
LS10	My physical recreational activities help me stay healthy.
LS11	I engage in recreational sports activities in places that interest me.

#### 2.2.3 Mental Toughness Scale

In order to align with the requirements of this study, the Sports Attitude Questionnaire (SAQ) from the previous study was revised. As shown in Table 3, the revised scale encompasses five dimensions: concentration, emotional control, positive cognition, family support, and interpersonal collaboration. The content validity of the questionnaire was also evaluated through an expert survey, and the results demonstrated a high level of validity (Nartova-Bochaver et al., 2021).

**Table 3:** Mental Toughness Scale.

Serial number	Measurement items
MT1	I can achieve my athletic goals.
MT2	I will not be discouraged by failure.
MT3	I like to seek new challenges in sports.
MT4	I feel empowered by the difficulties I encounter in sports.
MT5	I am able to concentrate and think clearly under the pressure of difficult situations.
MT6	I can make unusual or difficult decisions when developing an exercise program.
MT7	I can adapt to the internal and external changes that occur during exercise.
MT8	My past successes have given me the confidence to face the challenges of the sport.
MT9	After experiencing instances of competition or sports injuries, I tend to recover quickly.
MT10	When the movement is in trouble, I know where to go for help.
MT11	I feel in control of my athletic life.

#### 2.2.4 Self-Efficacy Scale

The concept of self-efficacy theory concerns an individual’s subjective evaluation of their capacity to perform a specific behavior. This evaluation, in turn, serves to enhance the individual’s motivation to act in a manner that leads to an improvement in their psychological state and self-confidence. The scale was developed by the German psychologist Ralph Schwarzer and his colleagues in 1981 and was selected for revision according to the requirements of this study, as shown in Table 4. It was subsequently used in the survey (Steigen et al., 2022).

**Table 4:** Self-Efficacy Scale.

Serial number	Measurement items
SE1	If I do my best, I’ll always be able to finish even the most difficult sports.
SE2	Even when people questioned me, I was able to stick to the sport I wanted to do.
SE3	For me, sticking to my ideals and reaching my athletic goals is a sure thing.
SE4	I’m confident that I can effectively deal with anything that comes out of the blue in sports.
SE5	With my talents, I’ll be able to handle the unexpected in sports.
SE6	If I put in the necessary effort, I’m bound to be able to solve most of the puzzles in sports.
SE7	I can face the difficulties of sports with equanimity because I believe in my ability to handle things.

### 2.3 Statistical analysis

IBM SPSS Statistics 26.0 was used for descriptive analysis of relevant variables, correlation analysis, and AMOS was also used to analyze the mediating effects of leisure satisfaction and mental toughness between exercise motivation and self-efficacy; Cronbach’s alpha coefficients and validation factor analyses were used for the reliability tests, respectively. In this study, a total scale containing all items was established, and in order to ensure that the internal consistency of the scale was guaranteed, the internal consistency reliability coefficients of the scale were tested for internal consistency with Cronbach’s *α* values before conducting the validation factor analysis. In the event that the correlation between the item and the dimension is low, and the overall reliability of the remaining items in the dimension is significantly enhanced following their removal, the item is deleted. In order to verify the structural model and data fitting, the following statistical indices were employed in the Structural Equation Modeling (SEM) analysis: Chi-square, df, Chi-square/df, GFI, CFI, and AGFI.

## 3 Results

### 3.1 Reliability and validity

As demonstrated in Table 5, the Cronbach’s *α* coefficient value of each variable exceeds 0.7, indicating that each measurement model exhibits good internal consistency reliability. Furthermore, the variables have been tested for validity. The factor loadings of all items are greater than 0.7, and the AVE values of the latent constructs are essentially greater than 0.5, indicating that each measurement model has good convergent validity. As illustrated in Table 6, with regard to discriminant validity, the square root of the AVE values of all potential constructs is greater than the correlation coefficient between the potential construct and any other potential construct, thereby indicating that each measurement model has good discriminant validity.

**Table 5:** Cronbach’s *α* values and factor loadings, CR and AVE for each item.

Variable	Item	Loadings	Cronbach’s *α*	CR	AVE
Exercise motivation	EM1	1.02	0.877	0.877	0.542
	EM2	1.02			
	EM3	1.02			
	EM4	0.91			
	EM5	0.99			
	EM6	1.00			
Leisure satisfaction	LS1	1.00	0.932	0.932	0.556
	LS2	0.95			
	LS3	0.88			
	LS4	0.85			
	LS5	0.95			
	LS6	0.92			
	LS7	0.90			
	LS8	0.86			
	LS9	0.90			
	LS10	0.92			
	LS11	0.95			
Mental toughness	MT1	1.07	0.929	0.929	0.542
	MT2	1.03			
	MT3	0.99			
	MT4	1.11			
	MT5	1.03			
	MT6	1.04			
	MT7	1.07			
	MT8	1.00			
	MT9	0.99			
	MT10	1.01			
	MT11	1.00			
Self-efficacy	SE1	1.00	0.897	0.893	0.544
	SE2	1.07			
	SE3	1.00			
	SE4	0.97			
	SE5	1.00			
	SE6	0.95			
	SE7	1.01			

**Table 6:** Discriminant validity of reflective measurement models.

	Exercise motivation	Leisure satisfaction	Mental toughness	Self-efficacy
Exercise motivation	**0.736**			
Leisure satisfaction	0.486	**0.746**		
Mental toughness	0.427	0.462	**0.736**	
Self-efficacy	0.488	0.608	0.54	**0.738**

The diagonal line is the square root of the AVE value for each potential construct, and the rest are the correlation coefficients between potential constructs.

### 3.2 Structural equation modeling analysis

**Figure 2:**
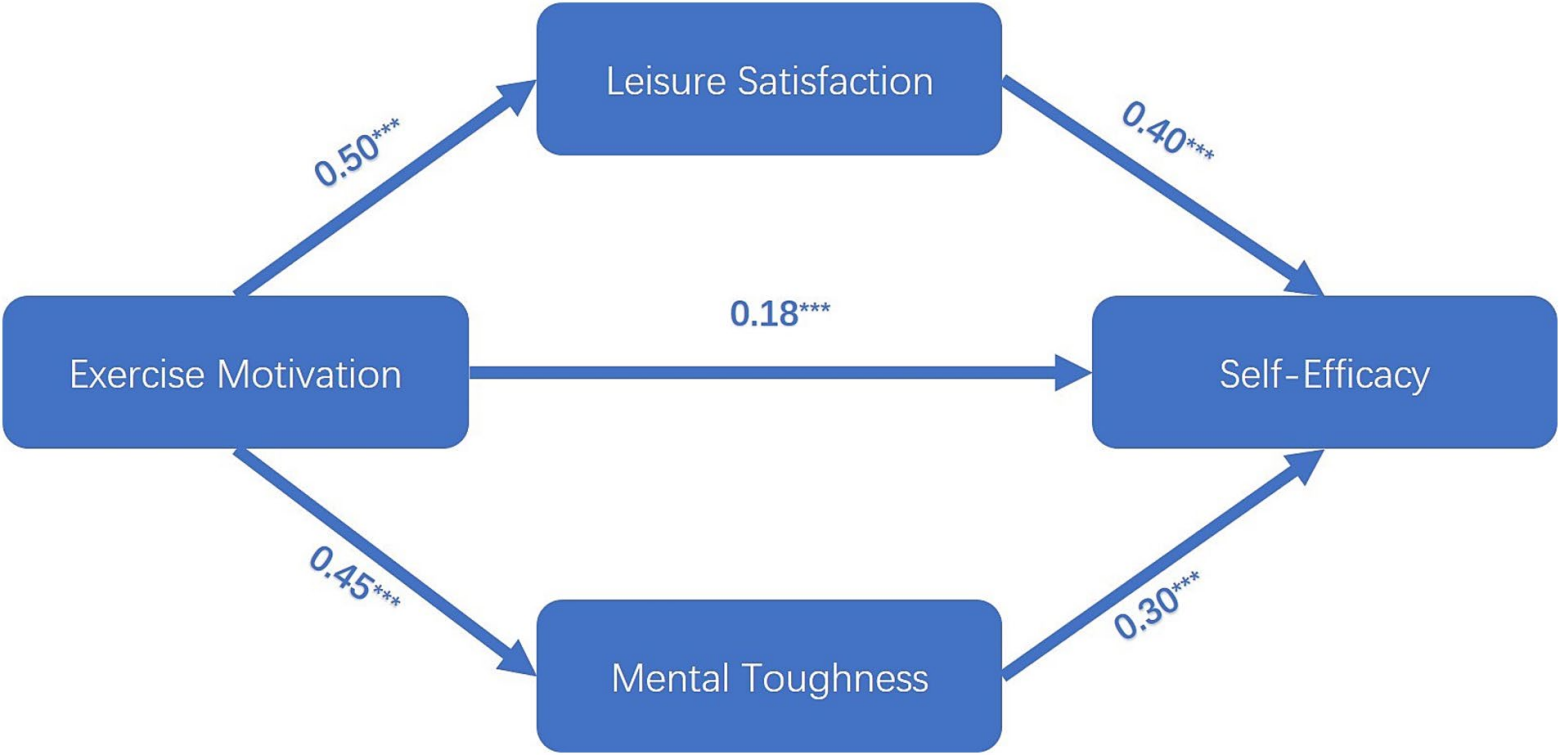
Effect of exercise motivation on self-efficacy through rest satisfaction and mental toughness (Chi-square = 924.131, df = 555, Chi-square/Df = 1.655, GFI = 0.932, CFI = 0.974, AGFI = 0.923). ***p < 0.001.

In this study, Amos data processing software was employed to ascertain the statistical significance of path coefficients. As illustrated in Figure 2, the findings revealed that exercise motivation exerted a statistical positive influence on self-efficacy (*β* = 0.18, *p* < 0.001) and on leisure satisfaction (*β* = 0.50, *p* < 0.001). Furthermore, exercise motivation was found to have a significant positive effect on mental toughness (*β* = 0.45, *p* < 0.001). Additionally, leisure satisfaction was observed to have a significant positive effect on self-efficacy (*β* = 0.40, *p* < 0.001), while mental toughness was also found to have a significant positive effect on self-efficacy (*β* = 0.30, *p* < 0.001). In light of the aforementioned evidence, it can be concluded that H1, H2, H3, H4, and H5 are supported.

As shown in Table 7, in terms of mediating effects, the direct, indirect, and total effects between exercise motivation, leisure satisfaction, mental toughness, and self-efficacy were measured using the mediating effects analysis procedure in the Amos data analysis software. First, in the path relationship “motivation → leisure satisfaction → self-efficacy,” the direct effect was significant, the indirect effect was significant, and there was a full mediation effect. In addition, in the path relationship ‘motivation → mental toughness → self-efficacy,’ the direct effect was significant, the indirect effect was significant, and there was a complete mediation effect. In conclusion, hypotheses H6 and H7 are supported.

**Table 7:** Results of the mediation effect.

Variable	Point estimate	Product of coefficient		Bootstrapping			
		SE	*Z*	Bias-corrected 95% CI		Percentile 95% CI	
				Lower	Upper	Lower	Upper
IE	0.33	0.039	0.001	0.26	0.409	0.261	0.409
SM → LS → SE	0.199	0.03	0.001	0.141	0.261	0.143	0.261
SM → RL → SE	0.131	0.027	0.001	0.086	0.194	0.084	0.189
DE	0.174	0.058	0.001	0.065	0.29	0.064	0.29
TE	0.504	0.053	0.001	0.399	0.607	0.401	0.611

## 4 Discussion

The aim of this study was to investigate the effects of exercise motivation on the self-efficacy of college students. To this end, a questionnaire survey was conducted among college students in Shandong Province, which revealed the mediating roles of leisure satisfaction and mental toughness. The results show that exercise motivation has a direct effect on self-efficacy, while leisure satisfaction and mental toughness play a significant mediating role in the relationship between exercise motivation and self-efficacy.

Motivation represents an intrinsic catalyst for an individual to accomplish a task and is the primary driver of individual behavior (Deci et al., 1991; Ryan and Deci, 2000). Those who are highly motivated to engage in physical activity tend to exhibit a heightened sense of self-efficacy, which can be defined as the belief in one’s ability to complete a task successfully (Boonekamp et al., 2020; Zhang et al., 2012; Jain et al., 2021). Individuals who engage in physical activity for its own sake are more likely to experience an enhanced sense of self-efficacy, which not only enables them to effectively complete their exercise regimen but also fosters a sense of self-efficacy and competence that extends beyond the immediate exercise session (Pekmezi et al., 2009). This is in accordance with Bandura’s (1997) self-efficacy theory, which posits that it is more straightforward to cultivate and sustain a high level of self-efficacy when engaging in activities that are enjoyable. It can be reasonably deduced that in order to successfully increase the number of individuals participating in exercise, it is necessary to place emphasis on enhancing their intrinsic motivation. This may be achieved by the implementation of diverse, engaging, and challenging exercise programs, which will stimulate individuals’ interest and enthusiasm. Concurrently, the development of individuals’ self-efficacy through the setting of small, achievable goals and the provision of positive feedback and support can also markedly enhance their motivation and participation (Plotnikoff et al., 2013). This study lends support to the positive correlation between exercise motivation and self-efficacy, thereby underscoring the pivotal role of intrinsic motivation and self-efficacy in fostering exercise behavior. These findings not only enhance the theoretical foundation of exercise psychology but also provide crucial guidance for practice, enabling the design of more effective exercise intervention strategies.

It is important to consider the role of leisure satisfaction in this study, and the results indicate that leisure satisfaction plays a significant mediating role in exercise motivation and self-efficacy. It has been previously highlighted that there is a positive correlation between exercise motivation and leisure satisfaction. However, no study has yet proposed that the pathway EM → LS → SE is viable. This study, therefore, represents a significant contribution to the field, confirming for the first time that exercise motivation can modulate self-efficacy through the mediating variable of leisure satisfaction. Consequently, while we focus on enhancing self-efficacy through exercise motivation, we can also consider the role of leisure satisfaction and mental toughness. It may be the case that a more optimal program can be identified. This is consistent with the findings of other researchers that self-efficacy moderates the relationship between task-oriented motivational climate and satisfaction. However, it differs from this paper in that collective efficacy mediates the relationship between baseline perceived motivational climate and satisfaction (Blecharz et al., 2014). Moreover, in modeling the mediation relationships, we acknowledge the potential for collinearity effects among the latent variables, particularly given the interdependence between exercise motivation, leisure satisfaction, and mental toughness. Although we were unable to compute specific metrics for multicollinearity in this study, we took care to design the study and select measures in a way that minimizes potential collinearity issues. Future research could benefit from a more detailed examination of this aspect to enhance the understanding of the relationships among these constructs.

In general, an individual’s motivation and beliefs regarding the completion of a specific task influence their actual performance and self-perception (Anderson et al., 2007). College students with higher motivation are more likely to feel competent in sports, which subsequently enhances their self-efficacy (Bandhu et al., 2024; Bandura, 1988). Exercise motivation encourages individuals to engage in physical activity and enhances their level of participation (Rahman et al., 2020). The acquisition of a sense of achievement and pleasure in sports has been demonstrated to increase self-efficacy. In particular, the attainment of goals in sports serves to reinforce this sense of achievement, which in turn enhances self-efficacy. These findings have significant implications for educational practice and mental health interventions, particularly in terms of enhancing students’ academic and life performance. Educators can enhance students’ self-efficacy by encouraging them to engage in physical activity. The implementation of diverse and challenging sports programs enables students to sustain their success and sense of control in sports, thereby enhancing their self-confidence and self-efficacy. Furthermore, college students who demonstrated higher levels of motivation exhibited enhanced leisure satisfaction and mental resilience following exercise (Yang and Qian, 2024; Choi et al., 2020). The observed increase in leisure satisfaction may be attributed to the sense of enjoyment and accomplishment associated with exercise (Wang, 2022). Similarly, the increase in mental toughness may be attributed to the process of encountering challenges and subsequently overcoming them during exercise. These findings are consistent with previous research, which has indicated that exercise has a beneficial effect on both physical and mental health, as well as on overall life satisfaction (Ryan and Deci, 2000). This finding emphasizes the importance of exercise in promoting overall well-being and psychological resilience, thereby reinforcing the necessity to integrate exercise into health promotion programs for college students. In addition, it was found that leisure satisfaction and psychological resilience have a significant positive effect on self-efficacy. It can be posited that elevated levels of leisure satisfaction may serve to augment positive affect and self-confidence, which in turn may lead to an increase in self-efficacy (Tian et al., 2022). Similarly, individuals who demonstrate higher levels of psychological resilience are more adept at coping with stress and challenges, which also contributes to their sense of self-efficacy (Wu et al., 2020; Qin et al., 2023). These findings lend support to the psychological capital theory put forth by Luthans et al. (2007), which posits that psychological resilience and self-efficacy are crucial elements of an individual’s positive psychological resources. This study lends further support to this theory, indicating that enhancing psychological resilience and leisure satisfaction represents an effective strategy for enhancing self-efficacy. It is of greater significance to note that our research has validated the mediating function of leisure satisfaction and mental toughness in the relationship between exercise motivation and self-efficacy. In particular, the motivation to exercise indirectly enhances self-efficacy by increasing individuals’ satisfaction with their leisure activities and psychological resilience. In line with the findings of Diotaiuti et al. (2023), which highlight the role of psychological mechanisms such as decentering in athletes, our study further explores how leisure satisfaction and mental toughness mediate the relationship between exercise motivation and self-efficacy in college students (Diotaiuti et al., 2023). This finding contributes to the theoretical frameworks of sports psychology and educational psychology by emphasizing the pivotal role of leisure satisfaction and mental toughness in the psychological development of individuals. The identification of these two variables as mediators allows us to elucidate the specific mechanisms through which exercise motivation affects self-efficacy. Furthermore, it provides clear targets for future interventions.

### 4.1 Limitations

This paper presents the findings of an investigation into the mechanism through which sports motivation exerts an influence on self-efficacy, which is mediated by leisure satisfaction and mental toughness. The investigation was conducted through a survey of college students who participate in sports in Shandong Province. This study is not without limitations. Firstly, although Shandong is one of China’s most populous provinces, the findings may not be representative of the wider Chinese population, potentially affecting the generalisability of the results. Secondly, the survey was conducted with a sample of college students, and therefore the findings cannot be generalized to the general public. Future research could consider longitudinal studies with larger sample sizes to further validate the relationship between sports motivation and self-efficacy. In conclusion, the cross-sectional design of our study precluded the establishment of causal relationships between variables. This is an inherent limitation of the analytical approach employed. To re-examine the causal relationships along each pathway, longitudinal or controlled trials are required.

## 5 Conclusion

The aim of this study was to examine the effect of exercise motivation on self-efficacy and to explore possible mechanisms. Our results indicate that exercise motivation has a significant positive effect on self-efficacy. Life satisfaction and mental toughness appear to be important mediators between exercise motivation and self-efficacy. One possible explanation is that students may use exercise as a tool to alleviate their poor physical and mental health.

## Data Availability

The raw data supporting the conclusions of this article will be made available by the authors, without undue reservation.
